# Effectiveness of burosumab in children with X-linked hypophosphatemia with low rickets severity: SUNFLOWER analysis

**DOI:** 10.1093/jbmrpl/ziag168

**Published:** 2026-10-15

**Authors:** Hee Gyung Kang, Toshimi Michigami, Noriyuki Namba, Nobuaki Ito, Takuo Kubota, Osamu Miyazaki, Masanori Kanematsu, Chikako Hayashi, Haruka Ishii, Seiji Fukumoto, Keiichi Ozono

**Affiliations:** Department of Pediatric Nephrology, Seoul National University Children’s Hospital, Seoul 03080, Republic of Korea; Department of Bone and Mineral Research, Osaka Women’s and Children’s Hospital, Osaka Prefectural Hospital Organization, Osaka 594-1101, Japan; Division of Pediatrics and Perinatology, Faculty of Medicine, Tottori University, Tottori 683-8503, Japan; Division of Therapeutic Development for Intractable Bone Diseases, Graduate School of Medicine and Faculty of Medicine, The University of Tokyo 113-0033, Tokyo, Japan; Department of Pediatric Nephrology and Metabolism, Osaka Women’s and Children’s Hospital, Osaka Prefectural Hospital Organization, Osaka 594-1101, Japan; Department of Radiology, National Center for Child Health and Development, Tokyo 157-8535, Japan; Medical Affairs Department, Kyowa Kirin Co., Ltd., Tokyo 100-0004, Japan; Medical Affairs Department, Kyowa Kirin Co., Ltd., Tokyo 100-0004, Japan; Medical Affairs Department, Kyowa Kirin Co., Ltd., Tokyo 100-0004, Japan; Tamaki-Aozora Hospital, Tokushima 779-3125, Japan; Center for Promoting Treatment of Intractable Diseases, ISEIKAI International General Hospital, Osaka 530-0052, Japan

**Keywords:** burosumab, phosphate metabolism, rare bone disease, rickets, X-linked hypophosphatemia

## Abstract

Burosumab provides treatment benefits in children with X-linked hypophosphatemia (XLH). However, children with low rickets severity (Rickets Severity Score [RSS] < 2) were excluded from a previous phase 3, multicenter, active comparator-controlled randomized clinical trial. This descriptive interim analysis of the prospective SUNFLOWER (Study of longitUdinal observatioN For patients with X-Linked hypOphosphatemic rickets/osteomalacia in collaboration With Asian partnERs) cohort study included 69 children with XLH aged ≤13 yr at enrollment. The burosumab and non-burosumab groups included 48 and 65 participants, respectively, with some overlap due to switching. Longitudinal changes in height and biochemical parameters (overall and stratified by disease severity [RSS <2/≥2; alkaline phosphatase </≥ upper limit of normal]) were assessed. In the burosumab all-patients analysis (irrespective of treatment duration or missing follow-up data), height *Z*-score (initiation: −2.04 ± 1.08; 3 yr: −1.58 ± 1.09), serum phosphate *Z*-score (initiation: −4.07 ± 1.77; 3 yr: −2.30 ± 1.45), 1,25(OH)_2_D (initiation: 39.79 ± 22.78 pg/mL; 3 yr: 84.35 ± 28.26 pg/mL), and ratio of tubular maximum phosphate reabsorption to glomerular filtration rate *Z*-score (initiation: −4.51 ± 2.60; 3 yr: −0.93 ± 2.50) tended to be higher at 3 yr following burosumab treatment. Rickets Severity Score (initiation: 2.38 ± 1.44; 3 yr: 0.83 ± 0.53) and alkaline phosphatase *Z*-score (initiation: 3.11 ± 1.79; 3 yr: 1.71 ± 1.14) tended to be lower at 3 yr. The subgroup analyses by RSS also showed similar results. Height *Z*-score, phosphate *Z*-score, and 1,25(OH)_2_D tended to be higher at 3 yr in both subgroups, while alkaline phosphatase *Z*-score tended to be lower. In the subgroup analysis by alkaline phosphatase status, height *Z*-score tended to be higher at 3 yr only in the ≥upper limit of normal subgroup; the other results were comparable. Overall, height, phosphate homeostasis, and bone health may improve in children with XLH (including RSS <2) following burosumab treatment.

## Teaser

**Lay Summary** X-linked hypophosphatemia (XLH) is a genetic condition characterized by low phosphate and active vitamin D, resulting in bone deformities, rickets, and poor growth in children. Burosumab provides treatment benefits to XLH patients. However, the benefits have mainly been demonstrated in children with more severe disease. We investigated whether burosumab benefits children with mild disease. We showed that burosumab tended to increase height, phosphate, and active vitamin D, and decrease rickets severity, in children with mild disease. Therefore, burosumab may benefit children with mild XLH.

## Graphical abstract

**Graphical abstract:**
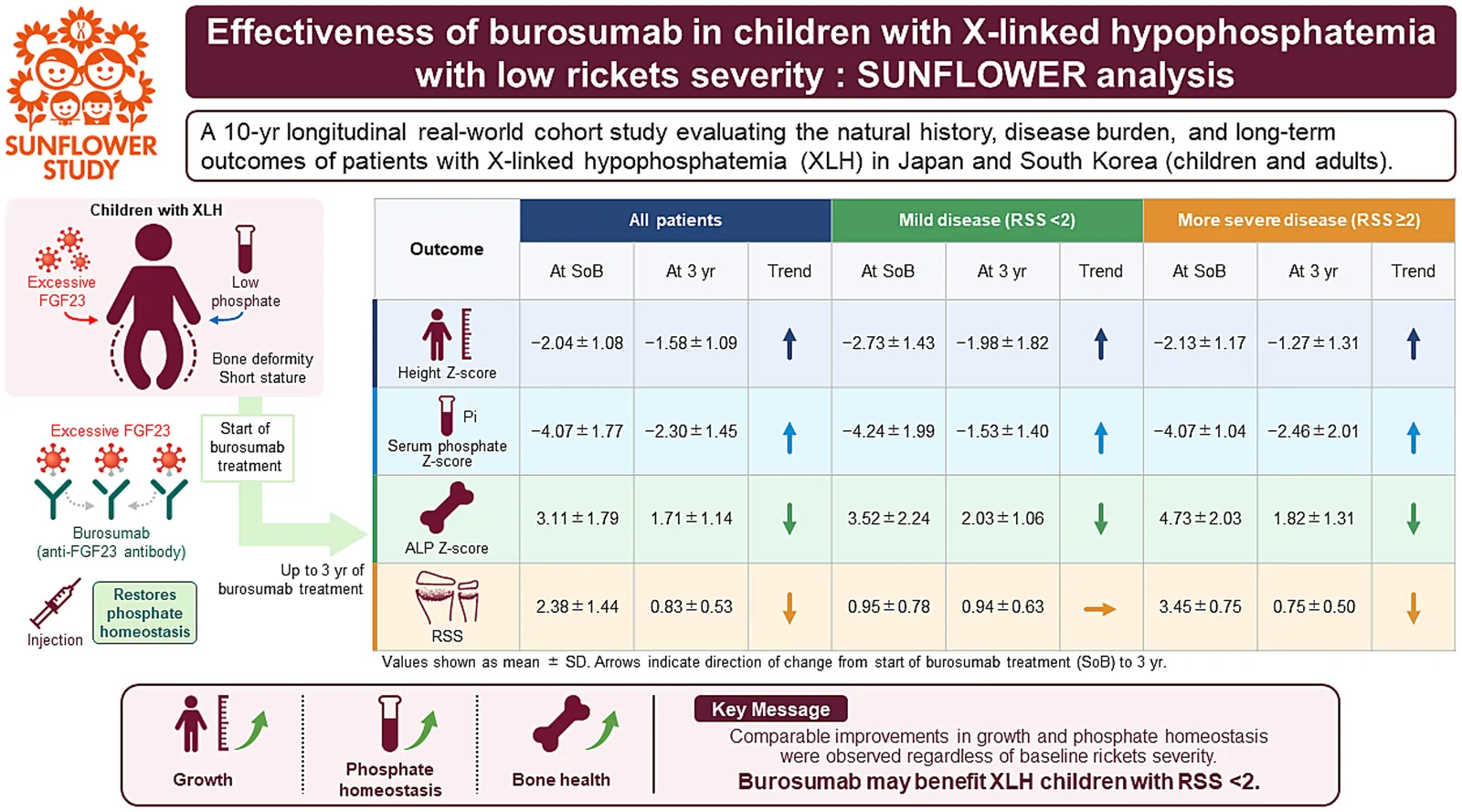

## Introduction

X-linked hypophosphatemia (XLH) is a rare genetic disorder with an estimated prevalence of 3.9-5 in 100 000 live births,^1^^,^^2^ although lower rates (1.3-1.7 in 100 000 live births) have been reported, perhaps because of misdiagnosis.^3–5^ X-linked hypophosphatemia results from loss-of-function variants in the phosphate-regulating endopeptidase homolog X-linked (*PHEX*) gene on the X chromosome.^6^^,^^7^

The PHEX protein is considered to regulate serum FGF23 levels because a loss or deficiency in PHEX function—such as that resulting from *PHEX* variants in XLH—increases FGF23 production,^8^ although the exact mechanism is unknown. Excess FGF23 reduces circulating 1,25(OH)_2_D by regulating the expression of enzymes involved in its activation and degradation. Specifically, excess FGF23 suppresses 25OHD-1α-hydroxylase (CYP27B1) expression, an enzyme responsible for the synthesis of 1,25(OH)_2_D, and induces 25OHD-24-hydroxylase (CYP24A1) expression, which catalyzes its degradation. The reduction in 1,25(OH)_2_D subsequently impairs intestinal absorption of calcium and phosphate, contributing to the pathophysiology of hypophosphatemic disorders. Additionally, FGF23 plays a critical role in maintaining phosphate homeostasis, particularly by regulating the expression of sodium-dependent phosphate co-transporters, namely NaPi-2a (SLC34A1) and NaPi-2c (SLC34A3), in the renal proximal tubules. Excess FGF23 leads to reduced intestinal phosphate absorption and increased urinary phosphate excretion, resulting in chronic hypophosphatemia.^9^

Chronic hypophosphatemia leads to defects in bone mineralization, rickets, short stature, and gait abnormalities in children, and fracture susceptibility, pain, and fatigue in adults, which are hallmarks of XLH.^8^^,^^10^ As such, XLH requires lifelong treatment. Patients with XLH have historically been treated with active vitamin D and oral phosphate preparations. However, these treatments have limitations, are associated with adverse effects (such as hyperparathyroidism and nephrocalcinosis^11^^,^^12^), and are poorly tolerated owing to gastrointestinal adverse effects and frequent dosing.^13–15^

Burosumab, a fully human monoclonal antibody that inhibits FGF23 activity, increases phosphate reabsorption in the kidneys and increases serum 1,25(OH)_2_D concentration.^16^ Burosumab was first launched for the treatment of XLH in the United States and Europe in 2018,^17^ and it was later launched in Japan in 2019^18^ and in South Korea in 2023.^19^ Clinical trials and a meta-analysis have demonstrated the benefits of burosumab in children with XLH, showing good safety and effectiveness.^20–23^ In recently published XLH treatment guidelines, burosumab is recommended as a first-line treatment for children with XLH.^24–27^ However, the availability of burosumab varies by country, with disease severity considered a condition for reimbursement in some countries.^28^^,^^29^

The Rickets Severity Score (RSS) and serum alkaline phosphatase (ALP) are used to evaluate XLH severity and monitor treatment response.^30^^,^^31^ A clinical trial showed that burosumab treatment in children with XLH with RSS ≥2 improved hypophosphatemia and reduced ALP, rickets, short stature, and defects in motor function, concluding that burosumab improved mineral and bone metabolism abnormalities.^32^ However, children with XLH with RSS <2 were excluded from the pivotal phase 3 trial.^32^^,^^33^ Therefore, there is limited clinical trial evidence on the usefulness of burosumab in children with XLH with RSS <2, and delays in optimal treatment initiation immediately after XLH diagnosis may result in suboptimal patient outcomes.

Considering the limited real-world evidence on the long-term effectiveness and safety of burosumab in pediatric patients with XLH, as well as the lack of evidence available for patients with RSS <2 and/or ALP below the upper limit of normal (ULN), we performed this 5-yr interim analysis of the ongoing 10-yr prospective SUNFLOWER (Study of longitUdinal observatioN For patients with X-Linked hypOphosphatemic rickets/osteomalacia in collaboration With Asian partnERs) study. Through this analysis, we aimed to describe the longitudinal changes (up to 3 yr) in the height and biochemical parameters of pediatric patients with XLH in the real-world setting, overall and stratified by disease severity (RSS <2 or RSS ≥2 and ALP < ULN or ALP ≥ ULN).

## Materials and methods

### Study design

The SUNFLOWER study is an ongoing prospective cohort study (up to 10 yr) conducted at 17 Japanese and 3 South Korean institutions. The study design has been described previously.^34^ The enrollment period was April 1, 2018, to April 30, 2022. The observation period started on April 1, 2018, and will end on April 30, 2028. The present report conveys the results of an interim analysis of data (cutoff December 2023), reporting the results of pediatric patients with XLH treated with burosumab for up to 3 yr.

### Ethics

The SUNFLOWER study is being conducted in compliance with the latest version of the Declaration of Helsinki and all relevant national regulations in Japan and South Korea. The ethics committees of the principal investigator’s medical institution (approval number: 17397 (T3)-18) and Kyowa Kirin Co., Ltd. (approval number: MA2023_012_11) approved the protocol and patient consent forms. The ethics committees of all other participating institutions also gave the necessary approvals. The SUNFLOWER study is registered in the University hospital Medical Information Network (UMIN000031605) established by the National University Hospital Council of Japan, and at ClinicalTrials.gov (NCT03745521). Written informed consent was obtained from the parents or legal guardians of the patients.

### Inclusion and exclusion criteria

In the SUNFLOWER study, patients with XLH were included if they met all of the following criteria: (1) had a confirmed *PHEX* gene mutation, a family member with an X-linked *PHEX* mutation, or an FGF23 concentration >30 pg/mL; (2) current or previous clinical or laboratory evidence of rickets or osteomalacia; and (3) written informed consent was obtained from the patient if aged ≥18 yr or from a parent or legal guardian if aged <18 yr. Patients were excluded if they were participating in another clinical trial at the time of obtaining consent, or if the principal investigator or sub-investigator judged them as unsuitable for participation.^34^

In this interim analysis, patients with XLH enrolled in the SUNFLOWER study were included if they were aged ≤13 yr at enrollment and had available data by the cutoff date (December 2023). If any patient reached 18 yr of age during the follow-up period, the data after the patient turned 18 yr of age were excluded. Patients aged >13 yr at enrollment or who started burosumab before enrollment were excluded.

### Treatments

Treatment selection was determined by the physician based on the patient’s condition and according to the treatments available in their country of residence.

In accordance with the package insert, burosumab was administered every 2 wk (pediatric regimen), or, for children with growth plate closure (as determined by the physician), every 4 wk (adult regimen).^35^ The initial dose was determined according to the patient’s body weight, with an initial dose for children of 0.8 mg/kg, which was increased or decreased according to the phosphate concentration and patient symptoms. For pediatric patients with XLH who were switched from the pediatric to the adult regimen, the initial burosumab dose was 1.0 mg/kg, and the dose was reduced according to the phosphate concentration and symptoms; no dose increases were used for these patients. All decisions regarding dosage and administration were made at the discretion of the attending physicians.

### Analysis populations

In the all-patients analysis, the burosumab group included patients who started burosumab after enrollment (during follow-up), while the non-burosumab group included patients who were not treated with burosumab at enrollment. When a patient started burosumab after enrollment, the data up to the point of starting burosumab were included in the non-burosumab group, and the data after starting burosumab were included in the burosumab group. If a patient discontinued burosumab, data up to the discontinuation date were analyzed.

We also performed a separate analysis of patients treated with burosumab or receiving non-burosumab treatment for the full 3 yr without missing data to evaluate outcomes when patients were treated and followed up for the full intended duration (full 3-yr analysis).

We conducted both analyses because each carries different bias risks. The all-patients analysis allows for the investigation of treatment choices in real-world clinical practice and minimizes selection bias. However, there is a risk of attrition bias owing to patients switching from the non-burosumab group to the burosumab group. The full 3-yr analysis, which excludes such transitions, is subject to selection and survival biases. Therefore, performing both analyses provided a more comprehensive understanding of the observed effects.

### Data analysis timepoints

In the burosumab group, data were analyzed at the start of burosumab administration (SoB; from 90 d before burosumab administration [day −90] to the day of administration [day 0]); and at 3 mo (days 1-150), 6 mo (days 151-240), 1 yr (days 241-425), 2 yr (days 606-790), and 3 yr (days 971-1155). In the non-burosumab group, data were analyzed at enrollment (from informed consent [day 0] to 90 d later [day 90]), and at 1 yr (days 241-425), 2 yr (days 606-790), and 3 yr (days 971-1155). Importantly, the time of initial data acquisition differed between the burosumab and non-burosumab groups. For the non-burosumab group, the initial data were obtained at enrollment, whereas for the burosumab group, the data were collected at burosumab initiation. The initial data shown for the non-burosumab group at enrollment also included pre-burosumab treatment data from patients in the burosumab group who had previously received non-burosumab therapy. As a result, there is a temporal discrepancy, with the burosumab baseline occurring later than the non-burosumab baseline. The flowchart of the all-patients analysis and the full 3-yr analysis in the burosumab and non-burosumab groups is shown in Figure S1.

Random blood and urine sampling was performed at least 4 h after a meal or intake of a phosphate preparation in principle. Disease-related complications, including new complications that occurred during follow-up, were recorded. Imaging data were used at each assessment point as appropriate, when available. The evaluations of RSS and Radiographic Global Impression of Change (RGI-C) were performed as described previously.^30^^,^^34^^,^^36^ Of note, for RSS/RGI-C, radiographic assessment was performed only when evaluable growth plates were present. Once epiphyseal plate closure was recognized on available radiographs or judged by the treating physician, RSS and RGI-C were no longer assessed, resulting in missing data rather than scores of 0.

### Subgroup analyses

Subgroup analyses were performed in the burosumab group, with the patients stratified by RSS (<2 or ≥2) and ALP (<ULN or ≥ULN) status. The ULN was based on age.^37^ In South Korea, it was expected that the number of patients with RSS <2 treated with burosumab would be extremely small because only XLH with RSS ≥2 is eligible for burosumab reimbursement. Therefore, we excluded patients from South Korea from the subgroup analyses by RSS and ALP status; the subgroup analyses were solely based on data from individuals from Japan.

### Primary outcome

To indicate how far height deviated from the average for age and sex, the change in height Z-score, calculated as described previously,^38^ from the SoB was reported as the primary outcome. As the sample size was expected to be small, no statistical tests were conducted; the results are descriptive only. Therefore, a numerical increase in height *Z*-score was considered to indicate a potential improvement in short stature.^32^ Continuous primary outcome variables are expressed as the mean ± SD.

### Secondary outcomes

The secondary outcomes included changes in RSS, serum ALP *Z*-score (calculated as described previously^39^), serum phosphate *Z*-score (calculated as described previously^40^), 1,25(OH)_2_D concentration, ratio of tubular maximum phosphate reabsorption to glomerular filtration rate (TmP/GFR) *Z*-score (calculated as described previously^39^), RGI-C, and serum intact PTH (iPTH) concentration. As the sample size was expected to be small, no statistical tests were conducted; the results are descriptive only. Numerical decreases in RSS and ALP, and numerical increases in phosphate, 1,25(OH)_2_D, TmP/GFR, and RGI-C from SoB were considered to indicate potential improvements.^32^ Continuous secondary outcome variables are expressed as the mean ± SD.

### Statistical analysis

The background characteristics in the burosumab group at the SoB and in the non-burosumab group at enrollment are expressed as summary statistics. Continuous background variables are expressed as the mean (95% CI); categorical variables are expressed as number (%). The non-burosumab and burosumab groups were not independent, making direct comparison of background characteristics between the groups inappropriate. All results are descriptive only. No sample size calculation was performed.

## Results

### Population

Overall, 226 patients participated in the SUNFLOWER study from April 1, 2018, to December 31, 2023. Among them, 147 were aged >13 yr at enrollment and 31 received burosumab before enrollment (with some overlap). These patients (*n* = 157) were excluded from this interim analysis, leaving 69 remaining patients. Among them, 65 and 4 patients were in the non-burosumab and burosumab groups, respectively, at enrollment. Overall, 44 patients in the non-burosumab group switched to the burosumab group between enrollment and the cutoff date. Therefore, 48 patients were analyzed in the burosumab group and 65 in the non-burosumab group. Among the 44 patients who started burosumab after enrollment, 35 started within 3 yr of enrollment, while 9 started >3 yr after enrollment. The patient flowchart is shown in Figure S2.

### Demographic characteristics at enrollment and at the SoB treatment

Table 1 summarizes the demographic characteristics of the groups in the all-patients analysis. The demographic characteristics of the non-burosumab group were based on the enrollment data, which included 44 patients who subsequently transitioned to the burosumab group out of a total of 65 patients. In contrast, the demographic characteristics of the burosumab group were defined as the data at the visit immediately before burosumab administration (SoB), which included 4 patients who initiated burosumab at the start of the study and 44 patients who started burosumab during follow-up, totaling 48 patients (Figure S2).

**Table 1:** Demographic and baseline characteristics of the burosumab and non-burosumab groups.

Characteristic		Burosumab (*n* = 48)	Non-burosumab (*n* = 65)
Sex (male)	*n* (%)	15 (31.2)	22 (33.8)
*PHEX* variant (positive)	*n* (%)	28 (58.3)	41 (63.1)
Age (yr)	Mean [95% CI]	8.64 [7.53, 9.76]	7.46 [6.50, 8.42]
Height (cm)	*n* ^†^	44	61
	Mean [95% CI]	117.25 [110.75, 123.75]	113.77 [108.25, 119.29]
Height (*Z*-score)	*n* ^†^	44	61
	Mean [95% CI]	−2.04 [−2.39, −1.69]	−1.88 [−2.17, −1.58]
Weight (kg)	*n* ^†^	46	64
	Mean [95% CI]	26.81 [23.44, 30.18]	23.89 [21.03, 26.74]
Weight (*Z*-score)	*n* ^†^	46	64
	Mean [95% CI]	−0.55 [−0.82, −0.27]	−0.48 [−0.71, −0.25]
Intact FGF23 (pg/mL)	*n* ^†^	46	59
	Mean [95% CI]	205.01 [154.73, −255.30]	249.80 [205.39, −294.20]
Phosphate (mg/dL)	*n* ^†^	46	61
	Mean [95% CI]	2.65 [2.45, 2.85]	2.98 [2.81, 3.16]
Phosphate (*Z*-score)	*n* ^†^	46	61
	Mean [95% CI]	−4.07 [−4.60, −3.54]	−3.57 [−4.03, −3.12]
Calcium (mg/dL)	*n* ^†^	46	61
	Mean [95% CI]	9.51 [9.41, 9.61]	9.51 [9.43, 9.60]
Albumin (g/dL)	*n* ^†^	46	61
	Mean [95% CI]	4.67 [4.60, 4.74]	4.57 [4.51, 4.63]
iPTH (pg/mL)	*n* ^†^	46	61
	Mean [95% CI]	43.89 [35.56, 52.22]	48.80 [41.57, 56.04]
1,25(OH)_2_D (pg/mL)	*n* ^†^	46	59
	Mean [95% CI]	39.79 [33.00, 46.58]	52.75 [46.75, 58.74]
25(OH)D (ng/mL)	*n* ^†^	46	61
	Mean [95% CI]	20.98 [18.48, 23.48]	22.95 [20.78, 25.12]
ALP (U/L)	*n* ^†^	46	61
	Mean [95% CI]	530.12 [466.53, 593.71]	514.61 [459.39, 569.83]
ALP (Z-score)	*n* ^†^	46	61
	Mean [95% CI]	3.11 [2.65, 3.57]	2.77 [2.37, 3.18]
Creatinine (mg/dL)	*n* ^†^	46	61
	Mean [95% CI]	0.34 [0.31, 0.37]	0.34 [0.31, 0.36]
TmP/GFR (mg/dL)	*n* ^†^	45	61
	Mean [95% CI]	2.38 [2.19, 2.57]	2.29 [2.13, 2.45]
TmP/GFR (*Z*-score)	*n* ^†^	45	61
	Mean [95% CI]	−4.51 [−5.60, −3.43]	−5.59 [−6.52, −4.67]
RSS average	*n* ^†^	18	35
	Mean [95% CI]	2.38 [1.75, 3.00]	1.86 [1.41, 2.31]
Surgical history	*n* (%)	4 (8.3)	0 (0.0)
Treatment at IC	*n* ^†^	44	65
Oral phosphate	*n* (%)	1 (2.3)	1 (1.5)
Active vitamin D	*n* (%)	2 (4.5)	2 (3.1)
Both	*n* (%)	40 (90.9)	60 (92.3)

Data in the burosumab group were obtained at the SoB treatment. Data in the non-burosumab group were obtained at enrollment. There is some overlap between the groups as some participants switched from non-burosumab treatment to burosumab. *n* indicates the number of patients, and *n*^†^ indicates the number of patients with available data.

Abbreviations: ALP, alkaline phosphatase; FGF23, fibroblast growth factor 23; IC, informed consent; iPTH, intact PTH; *PHEX*, phosphate-regulating endopeptidase homolog X-linked; RSS, Rickets Severity Score; SoB, start of burosumab; TmP/GFR, ratio of tubular maximum phosphate reabsorption to glomerular filtration rate.

The burosumab group had a numerically lower mean height *Z*-score (95% CI) than the non-burosumab group (−2.04 [−2.39, −1.69] and −1.88 [−2.17, −1.58], respectively). Mean RSS (2.38 [1.75, 3.00] and 1.86 [1.41, 2.31], respectively) and ALP *Z*-score (3.11 [2.65, 3.57] and 2.77 [2.37, 3.18], respectively) were numerically higher in the burosumab group, potentially reflecting more severe symptoms. The burosumab group had a numerically lower mean phosphate *Z*-score (−4.07 [−4.60, −3.54] and −3.57 [−4.03, −3.12], respectively) and 1,25(OH)_2_D (39.79 pg/mL [33.00, 46.58] and 52.75 pg/mL [46.75, 58.74], respectively) than the non-burosumab group. Interestingly, the burosumab group had a numerically higher mean TmP/GFR *Z*-score than the non-burosumab group (−4.51 [−5.60, −3.43] and −5.59 [−6.52, −4.67], respectively). There were no numerical differences in the iPTH concentration between the groups. As the two groups were not independent, statistical comparison was not performed.

### Burosumab group outcome analysis

The results of the all-patients analysis and the full 3-yr analysis are shown in Figure 1 and Table 2.

**Figure 1:**
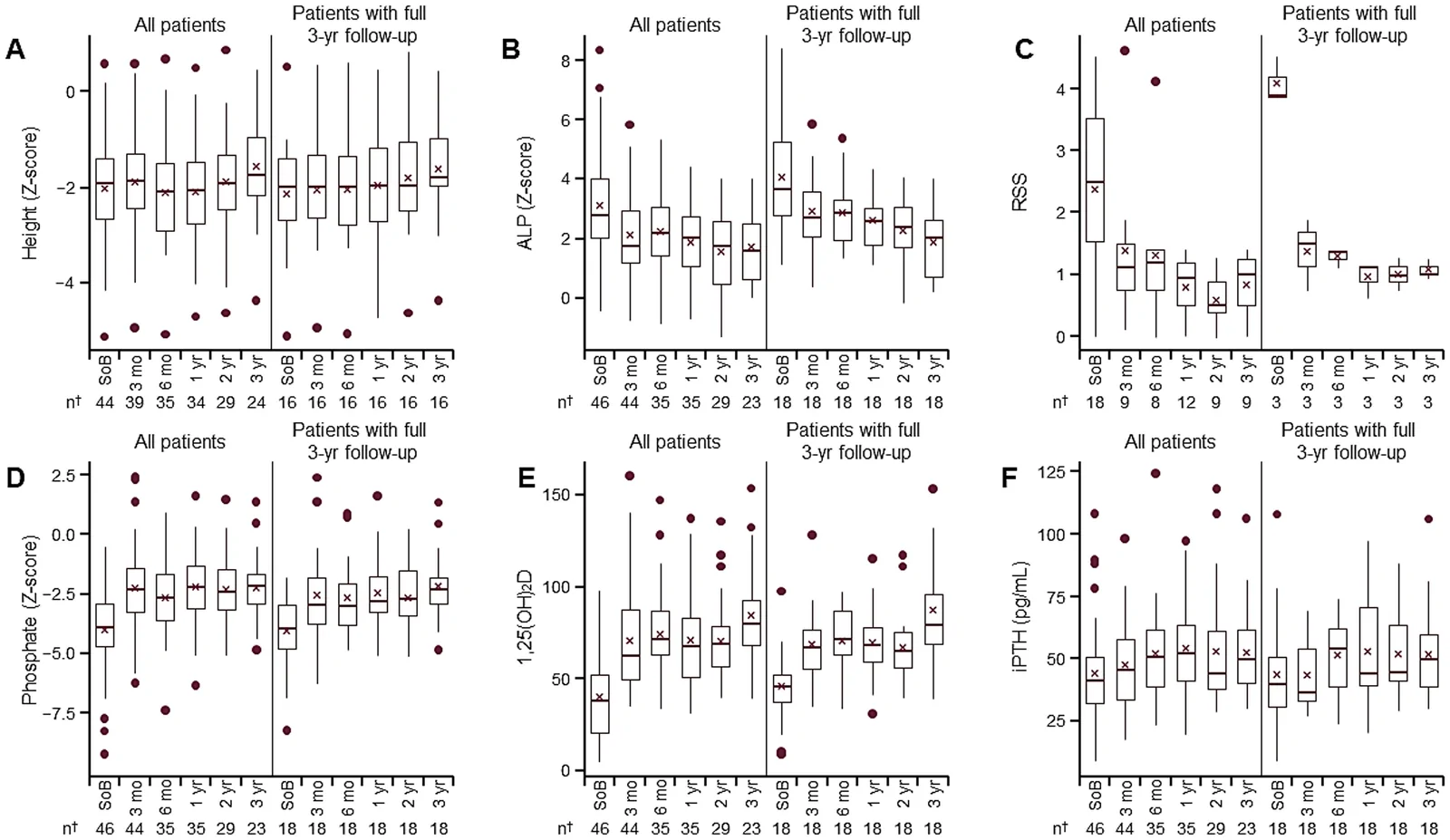
Results of the all-patients analysis and the full 3-yr analysis in the burosumab group. (A) Height *Z*-score, (B) ALP *Z*-score, (C) RSS, (D) phosphate *Z*-score, (E) 1,25(OH)_2_D, and (F) iPTH are shown at the SoB, 3 mo, 6 mo, 1 yr, 2 yr, and 3 yr for the all-patients analysis (irrespective of treatment duration and missing data) and the full 3-yr analysis (without missing data) in the burosumab group. The boxes represent the IQR, and the median is indicated by the horizontal line inside each box. The whiskers extend to the most extreme values within 1.5 × IQR. The mean value is indicated by a cross (×), and the circles represent outliers, defined as values outside the whiskers (1.5 × IQR). Here, *n*^†^ indicates the number of patients with available data. Abbreviations: ALP, alkaline phosphatase; iPTH, intact PTH; RSS, Rickets Severity Score; SoB, start of burosumab.

**Table 2:** Numerical changes from the SoB in the primary outcome measure in the burosumab group (all-patients analysis and full 3-yr analysis [without missing data]).

		All-patients analysis						Full 3-yr analysis					
		**SoB (*n* = 48)**	**3 mo (*n* = 48)**	**6 mo (*n* = 44)**	**1 yr (*n* = 38)**	**2 yr (*n* = 37)**	**3 yr (*n* = 33)**	**SoB (*n* = 33)**	**3 mo (*n* = 33)**	**6 mo (*n* = 33)**	**1 yr (*n* = 33)**	**2 yr (*n* = 33)**	**3 yr (*n* = 33)**
Height (*Z*-score)	*n* ^†^	44	39	35	34	29	24	16	16	16	16	16	16
	Mean ± SD	−2.04 ± 1.08	−1.91 ± 1.09	−2.13 ± 1.07	−2.10 ± 1.08	−1.91 ± 1.14	−1.58 ± 1.09	−2.13 ± 1.29	−2.05 ± 1.25	−2.04 ± 1.32	−1.95 ± 1.25	−1.79 ± 1.24	−1.59 ± 1.18

*n* indicates the number of patients, and *n*^†^ indicates the number of patients with available data.

Abbreviation: SoB, start of burosumab.

#### All-patients analysis

In the primary outcome analysis, mean height *Z*-score at 3 yr was numerically higher than at SoB (SoB: −2.04 ± 1.08; 3 yr: −1.58 ± 1.09). In the secondary outcome analysis, mean RSS at 3 yr was numerically lower than at the SoB (SoB: 2.38 ± 1.44; 3 yr: 0.83 ± 0.53). Mean RGI-C at 3 yr was 1.33 ± 0.29. Mean ALP *Z*-score at 3 yr was numerically lower than at the SoB (SoB: 3.11 ± 1.79; 3 yr: 1.71 ± 1.14). Conversely, mean phosphate *Z*-score (SoB: −4.07 ± 1.77; 3 yr: −2.30 ± 1.45), 1,25(OH)_2_D (SoB: 39.79 ± 22.78 pg/mL; 3 yr: 84.35 ± 28.26 pg/mL), and TmP/GFR *Z*-score (SoB: −4.51 ± 2.60; 3 yr: −0.93 ± 2.50) were higher at 3 yr. Although mean iPTH at 3 yr was numerically higher than at the SoB (SoB: 43.89 ± 20.30 pg/mL; 3 yr: 52.52 ± 17.53 pg/mL), iPTH was within the normal range at both timepoints (ULN = 65 pg/mL).

#### Full 3-yr analysis

In the primary outcome analysis, mean height *Z*-score tended to increase (SoB: −2.13 ± 1.29; 3 yr: −1.59 ± 1.18), similar to the all-patients analysis. In the secondary outcome analysis, mean RSS tended to decrease (SoB: 4.08 ± 0.36; 3 yr: 1.08 ± 0.14). Mean RGI-C at 3 yr was 1.33 ± 0.34. Consistent with the all-patients analysis, mean ALP *Z*-score tended to decrease (SoB: 4.04 ± 1.90; 3 yr: 1.86 ± 1.19). Mean phosphate *Z*-score (SoB: −4.09 ± 1.70; 3 yr: −2.18 ± 1.48), 1,25(OH)_2_D (SoB: 46.13 ± 21.66 pg/mL; 3 yr: 87.62 ± 29.11 pg/mL), and TmP/GFR *Z*-score (SoB: −4.68 ± 2.61; 3 yr: −0.69 ± 2.96) tended to increase, similar to the all-patients analysis. Although mean iPTH tended to increase (SoB: 43.39 ± 23.12 pg/mL; 3 yr: 51.61 ± 19.04 pg/mL), it was within the normal range at 3 yr, similar to the all-patients analysis.

### Non-burosumab group outcome analysis

The results of the all-patients analysis and the results of the full 3-yr analysis in the non-burosumab group are shown in Figure S3 and Table S1.

#### All-patients analysis

In the primary outcome analysis, mean height *Z*-score at 3 yr was numerically higher than at enrollment (enrollment: −1.85 ± 1.21; 3 yr: −1.25 ± 0.96). In the secondary outcome analysis, mean RSS at 3 yr was numerically higher than at enrollment (enrollment: 1.86 ± 1.27; 3 yr: 2.44 ± 0.80). Mean RGI-C at 3 yr was 1.33 ± 0.00, but the number of patients with analyzed data was only 2. Mean ALP *Z*-score at 3 yr was numerically lower than at enrollment (enrollment: 2.77 ± 1.39; 3 yr: 2.30 ± 1.29). Mean phosphate *Z*-score at 3 yr was similar to that at enrollment (enrollment: −3.57 ± 1.83; 3 yr: −3.45 ± 2.20). Moreover, mean 1,25(OH)_2_D (enrollment: 52.75 ± 23.56 pg/mL; 3 yr: 48.02 ± 29.81 pg/mL) was also similar to the value at enrollment. Mean TmP/GFR *Z*-score at 3 yr was numerically higher than at enrollment (enrollment: −5.59 ± 4.27; 3 yr: −4.25 ± 2.95). Mean iPTH fluctuated throughout the treatment period (enrollment: 48.80 ± 33.35 pg/mL; 1 yr: 61.51 ± 73.87 pg/mL; 2 yr: 52.28 ± 29.22 pg/mL; 3 yr: 40.44 ± 17.47 pg/mL).

#### Full 3-yr analysis

In the primary outcome analysis, there was no notable change in height *Z*-score (enrollment: −1.36 ± 1.04; 3 yr: −1.39 ± 0.86), even though the height *Z*-score at 3 yr was numerically higher than at enrollment in the all-patients analysis. In the secondary outcome analysis, similar to the all-patients analysis, there was no decrease in mean RSS at 3 yr (enrollment: 1.75 ± 0.88; 3 yr: 2.44 ± 0.80). Mean RGI-C at 3 yr was 1.33 ± 0.00, but the number of patients with analyzed data was only 2. Mean ALP *Z*-score tended to decrease (enrollment: 2.21 ± 1.22; 3 yr: 1.84 ± 1.10). There was no notable change in mean phosphate concentration (enrollment: 2.73 ± 0.82 mg/dL; 3 yr: 2.88 ± 0.66 mg/dL), although phosphate *Z*-score tended to increase (enrollment: −4.33 ± 2.56; 3 yr: −3.13 ± 2.38). There was no obvious change in mean 1,25(OH)_2_D (enrollment: 44.11 ± 20.62 pg/mL; 3 yr: 46.73 ± 16.90 pg/mL). A numerical increase in mean TmP/GFR *Z*-score was observed (baseline: −5.99 ± 4.57; 3 yr: −3.73 ± 2.63). Mean iPTH fluctuated throughout treatment (enrollment: 49.64 ± 33.95 pg/mL; 1 yr: 59.37 ± 43.81 pg/mL; 2 yr: 59.60 ± 33.22 pg/mL; 3 yr: 39.00 ± 12.93 pg/mL), similar to the all-patients analysis.

### Subgroup analyses

#### RSS status

The results of the burosumab subgroup analysis by RSS status at the SoB (RSS <2 or RSS ≥2) are shown in Figure 2 and Table 3 for the all-patients analysis and in Figure S4 for the full 3-yr analysis. The results were similar for both analyses. In the all-patients analysis, for the primary outcome, mean height *Z*-score at 3 yr was numerically higher than at the SoB in both subgroups (RSS <2: SoB: −2.73 ± 1.43, 3 yr: −1.98 ± 1.82; RSS ≥2: SoB: −2.13 ± 1.17, 3 yr: −1.27 ± 1.31). For the secondary outcome, mean RSS at 3 yr was numerically lower than at the SoB in the subgroup with RSS ≥2 (SoB: 3.45 ± 0.75; 3 yr: 0.75 ± 0.50), while the RSS at 3 yr was similar to at the SoB in the subgroup with RSS <2 (SoB: 0.95 ± 0.78; 3 yr: 0.94 ± 0.63). Mean RGI-C was similar at 3 yr in both subgroups (RSS <2: 1.33 ± 0.27, RSS ≥2: 1.33 ± 0.34). Mean ALP Z-score was numerically lower at 3 yr in both subgroups (RSS <2: SoB: 3.52 ± 2.24, 3 yr: 2.03 ± 1.06; RSS ≥2: SoB: 4.73 ± 2.03, 3 yr: 1.82 ± 1.31). Mean phosphate *Z*-score was higher at 3 yr in both subgroups (RSS <2: SoB: −4.24 ± 1.99, 3 yr: −1.53 ± 1.40; RSS ≥2: SoB: −4.07 ± 1.04, 3 yr: −2.46 ± 2.01). Mean 1,25(OH)_2_D was also numerically higher at 3 yr in both subgroups (RSS <2: SoB: 43.50 ± 23.97 pg/mL, 3 yr: 77.70 ± 10.44 pg/mL; RSS ≥2: SoB: 50.21 ± 11.77 pg/mL, 3 yr: 84.99 ± 23.69 pg/mL). Mean TmP/GFR *Z*-score was numerically higher at 3 yr in both subgroups (RSS <2: SoB: −5.09 ± 3.23, 3 yr: −0.76 ± 3.40; RSS ≥2: SoB: −5.18 ± 3.20, 3 yr: −0.38 ± 4.07). Mean iPTH in the RSS <2 subgroup was numerically higher at 3 yr (SoB: 46.29 ± 10.32 pg/mL, 3 yr: 59.50 ± 19.02 pg/mL), while it was slightly lower in the RSS ≥2 subgroup at 3 yr (SoB: 52.00 ± 27.48 pg/mL, 3 yr: 42.86 ± 8.34 pg/mL).

**Figure 2:**
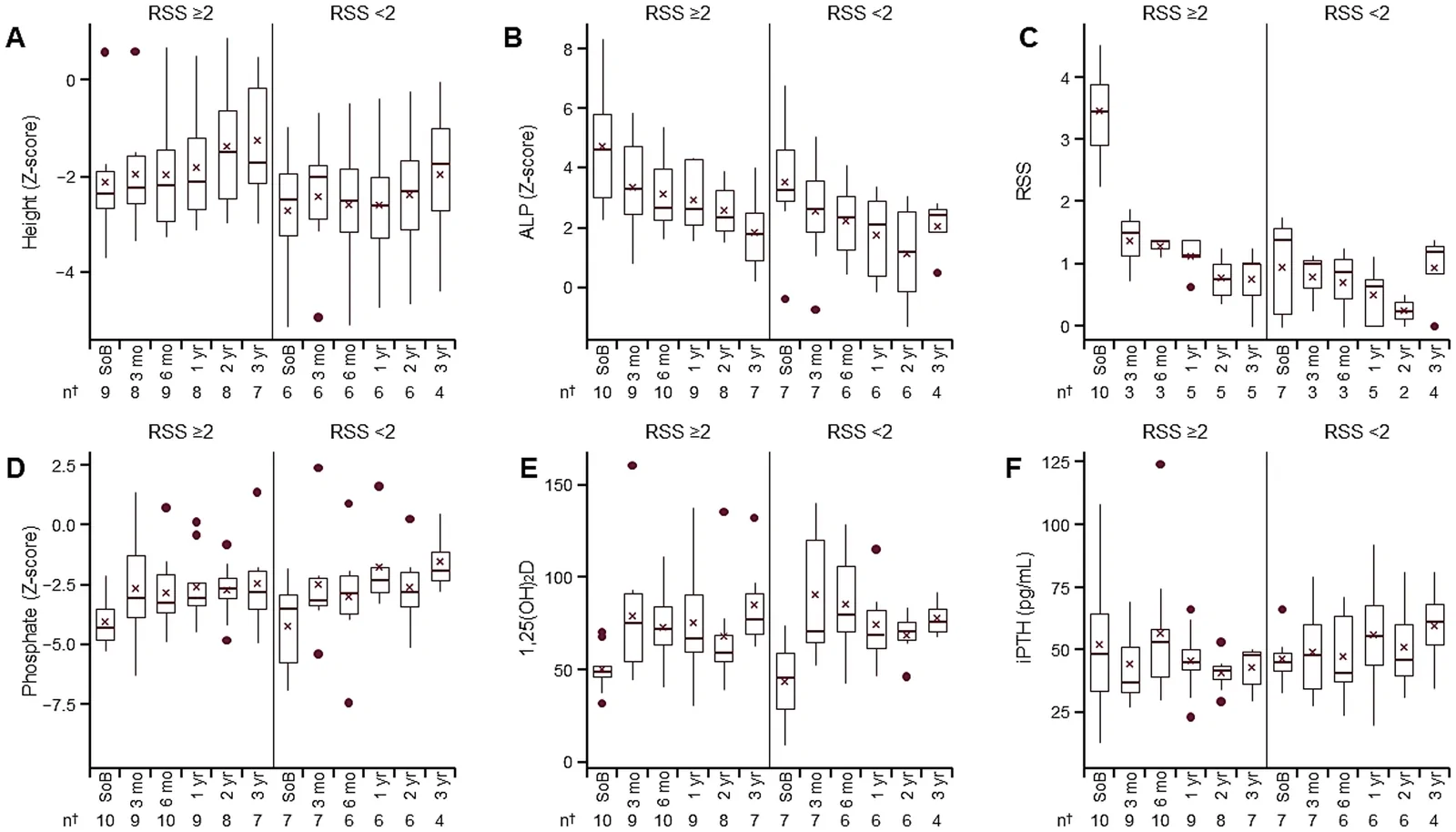
Results of the subgroup analysis by baseline RSS status (RSS <2 or RSS ≥2) in the burosumab group (all-patients analysis). (A) Height *Z*-score, (B) ALP *Z*-score, (C) RSS, (D) phosphate *Z*-score, (E) 1,25(OH)_2_D, and (F) iPTH are shown at the SoB, 3 mo, 6 mo, 1 yr, 2 yr, and 3 yr. the boxes represent the IQR, and the median is indicated by the horizontal line inside each box. The whiskers extend to the most extreme values within 1.5 × IQR. The mean value is indicated by a cross (×), and the circles represent outliers, defined as values outside the whiskers (1.5 × IQR). Here, *n*^†^ indicates the number of patients with available data. Abbreviations: ALP, alkaline phosphatase; iPTH, intact PTH; RSS, Rickets Severity Score; SoB, start of burosumab.

**Table 3:** Numerical changes from the SoB in the primary outcome measure in the burosumab group (all patients) stratified by baseline RSS (<2 or ≥2) and ALP (<ULN or ≥ULN).

		RSS < 2						RSS ≥ 2					
		**SoB (*n* = 7)**	**3 mo (*n* = 7)**	**6 mo (*n* = 7)**	**1 yr (*n* = 6)**	**2 yr (*n* = 6)**	**3 yr (*n* = 5)**	**SoB (*n* = 10)**	**3 mo (*n* = 10)**	**6 mo (*n* = 10)**	**1 yr (*n* = 10)**	**2 yr (*n* = 10)**	**3 yr (*n* = 9)**
Age at SoB (yr)	Mean ± SD	8.26 ± 5.19	-	-	-	-	-	6.90 ± 4.48	-	-	-	-	-
Height (*Z*-score)	*n* ^†^	6	6	6	6	6	4	9	8	9	8	8	7
	Mean ± SD	−2.73 ± 1.43	−2.43 ± 1.46	−2.61 ± 1.55	−2.61 ± 1.47	−2.40 ± 1.50	−1.98 ± 1.82	−2.13 ± 1.17	−1.96 ± 1.19	−1.98 ± 1.22	−1.82 ± 1.19	−1.39 ± 1.32	−1.27 ± 1.31
		**ALP < ULN**						**ALP ≥ ULN**					
		**SoB (*n* = 10)**	**3 mo (*n* = 10)**	**6 mo (*n* = 10)**	**1 yr (*n* = 10)**	**2 yr (*n* = 9)**	**3 yr (*n* = 7)**	**SoB (*n* = 28)**	**3 mo (*n* = 28)**	**6 mo (*n* = 27)**	**1 yr (*n* = 26)**	**2 yr (*n* = 26)**	**3 yr (*n* = 25)**
Age at SoB (yr)	Mean ± SD	11.33 ± 3.19	-	-	-	-	-	7.42 ± 3.90	-	-	-	-	-
Height (*Z*-score)	*n* ^†^	10	9	8	9	6	5	26	20	25	23	21	19
	Mean ± SD	−1.96 ± 1.14	−1.88 ± 1.22	−1.75 ± 0.93	−2.05 ± 1.12	−2.20 ± 0.94	−1.49 ± 0.64	−2.29 ± 1.08	−2.13 ± 1.16	−2.26 ± 1.13	−2.11 ± 1.12	−1.79 ± 1.21	−1.61 ± 1.19

*n* indicates the number of patients, and *n*^†^ indicates the number of patients with available data.

Abbreviations: ALP, alkaline phosphatase; RSS, Rickets Severity Score; SoB, start of burosumab; ULN, upper limit of normal.

#### ALP status

The results of the burosumab subgroup analysis by ALP status at the SoB are shown in Figure 3 and Table 3 for the all-patients analysis and are summarized in Figure S5 for the full 3-yr analysis. The results were similar for both analyses. In the all-patients analysis, for the primary outcome, mean height *Z*-score at 3 yr in the subgroup with ALP ≥ ULN was numerically higher than at the SoB (SoB: −2.29 ± 1.08, 3 yr: −1.61 ± 1.19), while in the subgroup with ALP < ULN, mean height *Z*-score was similar or slightly higher at 3 yr than at the SoB (SoB: −1.96 ± 1.14, 3 yr: −1.49 ± 0.64). A sensitivity analysis evaluating longitudinal height changes within the same individuals revealed that height *Z*-score in the subgroup with ALP < ULN tended to slightly increase over 3 yr of burosumab treatment (SoB: −1.73 ± 0.40; 3 yr: −1.57 ± 0.23). For the secondary outcome analysis, there were no RSS data at 3 yr in the ALP < ULN subgroup, so this was not analyzed. Mean RSS at 3 yr in the ALP ≥ ULN subgroup was numerically lower than at the SoB (SoB: 2.57 ± 1.37, 3 yr: 0.83 ± 0.53). The ALP *Z*-score at 3 yr was numerically lower than at the SoB only in the ALP ≥ ULN subgroup (ALP < ULN: SoB: 1.20 ± 0.64, 3 yr: 1.21 ± 0.97; ALP ≥ ULN: SoB: 4.01 ± 1.61, 3 yr: 1.82 ± 1.17). Mean phosphate *Z*-score was numerically higher at 3 yr in both subgroups (ALP < ULN: SoB: −3.61 ± 2.15, 3 yr: −1.86 ± 0.96; ALP ≥ ULN: SoB: −3.92 ± 1.43, 3 yr: −2.39 ± 1.53). Mean 1,25(OH)_2_D was also numerically higher at 3 yr in both subgroups (ALP < ULN: SoB: 33.46 ± 29.63 pg/mL, 3 yr: 96.55 ± 37.60 pg/mL; ALP ≥ ULN: SoB: 47.33 ± 19.43 pg/mL, 3 yr: 81.78 ± 26.47 pg/mL). Moreover, mean TmP/GFR Z-score was numerically higher in both subgroups at 3 yr (ALP < ULN: SoB: −2.76 ± 1.39, 3 yr: −0.78 ± 1.14; ALP ≥ ULN: SoB: −4.99 ± 2.81, 3 yr: −0.97 ± 2.74). Mean iPTH was numerically higher at 3 yr than at SoB in the ALP < ULN subgroup (SoB: 36.00 ± 21.45 pg/mL, 3 yr: 64.25 ± 29.34 pg/mL), whereas it was similar in the ALP ≥ ULN subgroup (ALP ≥ ULN: SoB: 48.46 ± 21.35 pg/mL, 3 yr: 50.05 ± 13.97 pg/mL). Radiographic Global Impression of Change was evaluated only in the ALP ≥ ULN subgroup owing to the lack of available data, and the mean RGI-C at 3 yr was 1.33 ± 0.29.

**Figure 3:**
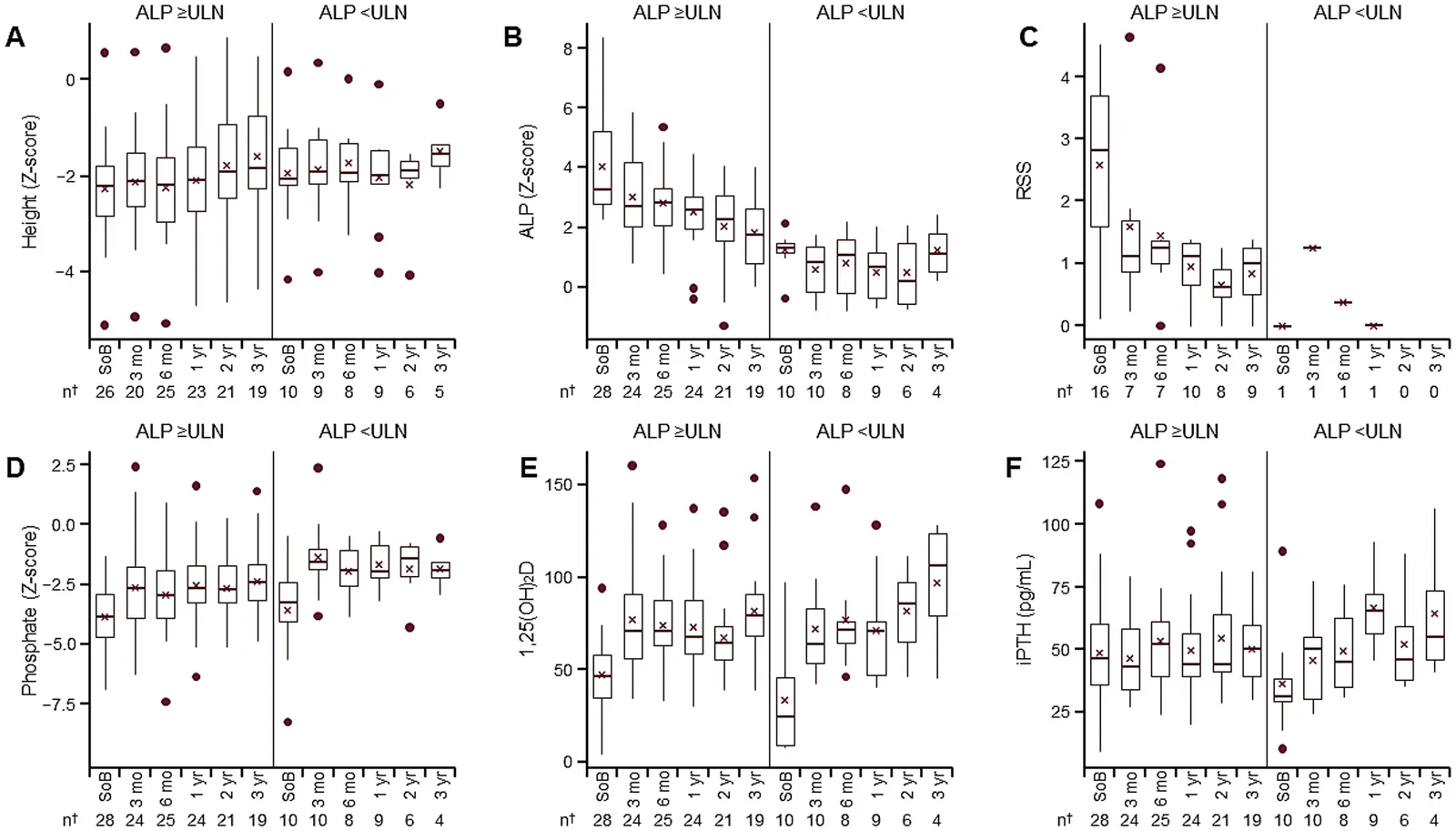
Results of the subgroup analysis by ALP status (ALP < ULN or ALP ≥ ULN) in the burosumab group (all-patients analysis). (A) Height *Z*-score, (B) ALP *Z*-score, (C) RSS, (D) phosphate *Z*-score, (E) 1,25(OH)_2_D, and (F) iPTH are shown at the SoB, 3 mo, 6 mo, 1 yr, 2 yr, and 3 yr. the boxes represent the IQR, and the median is indicated by the horizontal line inside each box. The whiskers extend to the most extreme values within 1.5 × IQR. The mean value is indicated by a cross (×), and the circles represent outliers defined as values outside the whiskers (1.5 × IQR). Here, *n*^†^ indicates the number of patients with available data. Abbreviations: ALP, alkaline phosphatase; iPTH, intact PTH; RSS, Rickets Severity Score; SoB, start of burosumab; ULN, upper limit of normal.

#### Safety

In the all-patients analysis, the mean renal calcification grade following burosumab administration was 0.86 ± 1.10 (*n* = 14) at SoB and 1.50 ± 1.00 (*n* = 4) at 3 yr. During burosumab treatment, 6 serious adverse events were observed: craniosynostosis, lower eyelid entropion (bilateral), intermittent exotropia (monocular), inguinal hernia (right), genu varum (for postoperative implant removal), and genu valgum (bilateral). Genu varum onset occurred before burosumab administration and was treated surgically; subsequent removal of the postoperative implant occurred after burosumab initiation and was therefore recorded during the treatment period. All events received appropriate management, including surgical intervention where necessary, and the condition of the patients improved.

## Discussion

This interim analysis of the SUNFLOWER study produced several noteworthy findings. A numerical increase in height *Z*-score, indicating an improvement in short stature, and numerical decreases in RSS and ALP *Z*-score were observed following burosumab treatment for up to 3 yr. Phosphate homeostasis appeared to improve, with numerical increases in phosphate *Z*-score, 1,25(OH)_2_D, and TmP/GFR *Z*-score, following burosumab treatment. These changes were observed even in patients with XLH with RSS <2, indicating that burosumab may have beneficial effects even in patients with low rickets severity, which has been poorly investigated previously.^20^^,^^41^ In the burosumab subgroup analysis by ALP status, numerical improvements in short stature and bone health were more apparent in patients who had ALP ≥ ULN at SoB.

Although numerically higher height *Z*-score and lower ALP *Z*-score were observed after 3 yr in the all-patients analysis in the non-burosumab group, in the full 3-yr analysis excluding missing data, these tendencies disappeared. Additionally, among the 65 patients enrolled in the non-burosumab group, 44 transitioned to the burosumab group (Figure S2). The reason for switching from the non-burosumab to burosumab group was likely lack of an improvement in the non-burosumab group. Furthermore, in the full 3-yr analysis, the non-burosumab group included patients who did not require burosumab therapy throughout the 3-yr period, and thus tended to include those with milder symptoms. As this group also had a higher height *Z*-score at enrollment than at the SoB, there may have been limited potential for further height improvement. The imbalance in the patient populations should be considered.

### Height

Numerical height improvement was observed following 3 yr of burosumab in the all-patients analysis and in the full 3-yr analysis, as well as in the subgroup analysis stratified by RSS status. The mean change in height *Z*-score at 3 yr was +0.46 overall, +0.86 in the RSS ≥2 subgroup, and +0.75 in the RSS <2 subgroup. The effects of burosumab on height in pediatric patients with XLH are corroborated by an open-label phase 3 trial of pediatric patients with XLH aged 1-12 yr with RSS ≥2.^22^ In that study, the burosumab group demonstrated an increase in height *Z*-score. The mean change at 64 wk was +0.36, and the mean change at 88 wk was +0.41.^22^ The numerical improvements observed in our study were consistent with the previous report, except we expanded the findings by including patients with RSS <2. Overall, the findings suggest that burosumab may improve short stature in pediatric patients with XLH under real-world clinical conditions.

In the SUNFLOWER study, many patients with XLH had been treated with oral phosphate/active vitamin D preparations before starting burosumab.^19^^,^^42^ Even patients with XLH with RSS <2 at baseline whose symptoms were well-controlled with oral phosphate and/or active vitamin D demonstrated numerical improvements in height *Z*-score after switching. One possible reason for the improvement in patients with RSS <2 is that the baseline height *Z*-score in the RSS <2 subgroup was −2.73 ± 1.43, lower than that in the RSS ≥2 subgroup (−2.13 ± 1.17). This lower baseline height may have provided greater potential for improvement. Although it is necessary to consider that patient characteristics at the SoB were not adjusted in the present study, the findings of this interim analysis suggest that short stature may improve in pediatric patients with XLH following burosumab treatment, even in patients with RSS <2.

The patients were also stratified by ALP status. At 3 yr, the mean height *Z*-score was numerically higher than at SoB in the ALP ≥ ULN subgroup (+0.68) and in the ALP < ULN subgroup (+0.47), although in the ALP < ULN subgroup, it fluctuated over the 3-yr period, and no consistent increasing pattern was observed throughout the entire 3-yr period. This may be because the mean age at baseline in the ALP < ULN and ALP ≥ ULN subgroups was 11.33 ± 3.19 yr and 7.42 ± 3.90 yr, respectively, and the degree of short stature according to height *Z*-score was milder in those in whom ALP had normalized before the SoB than in the subgroup with high ALP (SoB: ALP < ULN: −1.96 ± 1.14; ALP ≥ ULN: −2.29 ± 1.08), potentially preventing a pronounced increase in height from being detected in the ALP < ULN subgroup. It is also possible that patients with better height *Z*-score in the ALP < ULN subgroup were lost to follow-up and that only patients with shorter stature remained at 3 yr. This may have led to an apparent lack of improvement. In the sensitivity analysis evaluating longitudinal height changes within the same individuals, height *Z*-score tended to slightly increase over 3 yr with burosumab.

Although patients aged >13 yr at enrollment were excluded from the present study, some may have approached or reached epiphyseal closure during the 3-yr follow-up period; thus, some patients with closed growth plates may have been included. We acknowledge that including adolescents with possible epiphyseal closure may limit the interpretation of height *Z*-score changes. Nevertheless, a previous study reported continued height gains in adolescents aged 14-17 yr.^43^ Therefore, adolescents were retained in the primary descriptive analysis.

### RSS and RGI-C

There was a numerical improvement in RSS from the SoB (2.38 ± 1.44) to 3 yr (0.83 ± 0.53). In an open-label phase 3 trial,^22^ total RSS decreased from the SoB (3.08 ± 0.49) to week 64 (0.92 ± 0.49) and week 88 (0.92 ± 0.38) among pediatric patients with XLH treated with burosumab, indicating reduced rickets severity, corroborating the findings of the present study. However, the follow-up was shorter than the 3-yr follow-up in the present study, so our findings have additive value.

In the burosumab subgroup analysis by RSS status, RSS tended to decrease in the subgroup with RSS ≥2. In the subgroup with RSS <2, a decreasing tendency was observed up to 2 yr; however, this was not apparent at 3 yr, likely because the RSS was already low in the subgroup with RSS <2, preventing any further reduction following burosumab treatment. The sample size for the RSS <2 subgroup may have been insufficient for full 3-yr stratified analysis. Moreover, in the all-patients analysis, different individuals may have been assessed at each timepoint owing to missing data, potentially influencing the results.

The RGI-C is another reliable and sensitive measure in pediatric XLH, and is complementary to the RSS for the assessment of rickets severity.^44^ In the present study, burosumab marginally increased the RGI-C in the all-patients analysis. Notably, RGI-C increased to a similar level in both RSS subgroups (around 1.33) by 3 yr, indicating an improvement toward healing in both subgroups at a level between minimal (+1) and substantial (+2) based on the RGI-C 7-point scale.^45^ This finding is inconsistent with the RSS. One explanation is that RSS and RGI-C assess different anatomical sites and use different evaluation criteria. While these two methods are complementary, they do not always yield identical results.^44^ Comprehensive assessment of the results from both RSS and RGI-C possibly indicates an improvement in rickets severity even in patients with RSS <2 following burosumab treatment. These findings suggest that the RSS may decrease and the RGI-C increase following burosumab treatment, thus improving rickets in children with XLH in real-world settings.

### ALP normalization

One treatment goal of XLH is ALP normalization according to International and European recommendations for XLH treatment.^24^^,^^27^ However, ALP normalization with oral phosphate and/or active vitamin D preparations is challenging even when ALP normalization is achieved using these treatments, which may lead to adverse events such nephrocalcinosis or hyperparathyroidism due to excessive dosing,^11^ requiring careful monitoring.

Numerical improvement in ALP *Z*-score was observed following 3 yr of burosumab treatment in the all-patients analysis, full 3-yr analysis, and subgroups stratified by RSS status. In the subgroup analysis by ALP status, 28 of 38 patients (73.7%) did not achieve ALP normalization before burosumab therapy. In the burosumab group stratified by ALP status, among patients with ALP ≥ ULN, ALP *Z*-score tended to decrease over 3 yr from the SoB (4.01 ± 1.61) to 3 yr (1.82 ± 1.17). The numerical reduction in ALP *Z*-score in the ALP ≥ ULN subgroup is promising and may suggest that burosumab can normalize ALP. However, in the ALP ≥ ULN subgroup, ALP did not decrease to the value seen in the ALP < ULN subgroup at the SoB (1.20 ± 0.64). As there were also numerical improvements in short stature and bone health following burosumab in patients with ALP ≥ ULN, our findings are consistent with the treatment strategies recommended in current guidelines, which aim for ALP normalization.

Among the patients, 26.3% exhibited ALP normalization before burosumab administration, and no further decrease in ALP was observed after starting burosumab. Of the 10 patients with normalized ALP at the SoB, medication records were available for 9. Among them, 8 (88.9%) had been treated with both oral phosphate preparations and active vitamin D analogs. Numerical improvement in phosphate homeostasis was observed following burosumab administration. Long-term treatment with oral phosphate and/or active vitamin D can be associated with adverse effects, such as secondary hyperparathyroidism and nephrocalcinosis, as well as adherence issues. Therefore, burosumab may be considered for patients who have difficulty tolerating oral phosphate and active vitamin D.

### Phosphate, 1,25(OH)_2_D, and TmP/GFR

Phosphate *Z*-score and 1,25(OH)_2_D tended to increase following burosumab treatment in the all-patients analysis and in the subgroups stratified by RSS and ALP status. Moreover, TmP/GFR *Z*-score tended to increase following burosumab treatment, which is a crucial indicator of renal phosphate reabsorption. Similar results regarding phosphate and 1,25(OH)_2_D have been observed in a clinical trial.^32^ Therefore, our findings are corroborated by previous research.

### Safety

In all groups, mean iPTH at 3 yr was within the normal range (ULN = 65 pg/mL). Burosumab demonstrated an acceptable safety profile, with only a small number of mild and transient adverse events.

### Limitations

This study had some limitations. First, there was an imbalance in some background characteristics between groups, and no adjustment for baseline imbalance was performed. Additionally, the non-burosumab and burosumab groups were not independent, preventing direct comparison of background characteristics. Second, in the all-patients analysis, burosumab became commercially available during follow-up, which resulted in some patients switching from the non-burosumab to burosumab group, introducing the possibility of attrition bias in the all-patients analysis. Meanwhile, in the full 3-yr analysis, survivor bias or selection bias may exist. As both biases are critical, the analyses were conducted in both datasets. Third, because of switching from non-burosumab to burosumab, the data in the burosumab group were collected later than the data in the non-burosumab group for each patient, leading to a temporal discrepancy in data collection. Moreover, there were differences in the duration of therapy between the patients. Fourth, the follow-up intervals differed between the groups, with the former undergoing more frequent follow-up. Therefore, non-burosumab treatment effects may not have been captured in as much detail as those of burosumab. Fifth, as this was an observational study, radiographic data were not available for all patients at all assessment time points, and epiphyseal closure was not systematically recorded as a prespecified variable. Therefore, we could not reliably identify all patients with closed growth plates or exclude them from the height *Z*-score analysis. Given these limitations, we cannot rule out that the inclusion of patients with epiphyseal closure may have contributed to an underestimation of growth outcomes.

Finally, owing to the small sample size, we did not perform statistical comparisons. The data are intended to provide early descriptive insights into the results of the full SUNFLOWER study, which will be useful to determine significant differences between burosumab and non-burosumab therapy in pediatric patients with XLH in the real-world setting, particularly those stratified by disease severity according to RSS and ALP status.

### Conclusion

Among pediatric patients with XLH, numerical improvements in short stature, numerical reductions in rickets severity, a trend toward ALP normalization, and numerical improvements in phosphate homeostasis and bone health were observed following 3 yr of burosumab. These effects were observed even in those with RSS <2 before burosumab treatment.

## Supplementary Material

Supplementary_Materials_ziag168

## Data Availability

Research data, including participant data, the statistical analysis plan, and informed consent forms, are not shared. The study protocol has been published previously.^33^

## Clinical trial registration

NCT03745521; UMIN000031605.

## References

[1] Endo I, Fukumoto S, Ozono K, et al. Nationwide survey of fibroblast growth factor 23 (FGF23)-related hypophosphatemic diseases in Japan: prevalence, biochemical data and treatment. Endocr J. 2015;62(9):811–816. 10.1507/endocrj.EJ15-0275 26135520

[2] Beck-Nielsen SS, Brock-Jacobsen B, Gram J, Brixen K, Jensen TK. Incidence and prevalence of nutritional and hereditary rickets in southern Denmark. Eur J Endocrinol. 2009;160(3):491–497. 10.1530/EJE-08-0818 19095780

[3] Rafaelsen S, Johansson S, Ræder H, Bjerknes R. Hereditary hypophosphatemia in Norway: a retrospective population-based study of genotypes, phenotypes, and treatment complications. Eur J Endocrinol. 2016;174(2):125–136. 10.1530/EJE-15-0515 PMC4674593 26543054

[4] Hawley S, Shaw NJ, Delmestri A, et al. Prevalence and mortality of individuals with X-linked hypophosphatemia: a United Kingdom real-world data analysis. J Clin Endocrinol Metab. 2020;105(3):e871–e878. 10.1210/clinem/dgz203 PMC7025948 31730177

[5] Sandy JL, Nunez C, Wheeler BJ, et al. Prevalence and characteristics of paediatric X-linked hypophosphataemia in Australia and New Zealand: results from the Australian and the New Zealand paediatric surveillance units survey. Bone. 2023;173:116791. 10.1016/j.bone.2023.116791 37263386

[6] Ishihara Y, Ohata Y, Takeyari S, et al. Genotype–phenotype analysis, and assessment of the importance of the zinc-binding site in PHEX in Japanese patients with X-linked hypophosphatemic rickets using 3D structure modeling. Bone. 2021;153:116135. 10.1016/j.bone.2021.116135 34333162

[7] Yamamoto A, Nakamura T, Ohata Y, Kubota T, Ozono K. Phenotypes of a family with XLH with a novel *PHEX* mutation. Hum Genome Var. 2020;7:8. 10.1038/s41439-020-0095-1 PMC7109063 32257293

[8] Beck-Nielsen SS, Mughal Z, Haffner D, et al. FGF23 and its role in X-linked hypophosphatemia-related morbidity. Orphanet J Rare Dis. 2019;14(1):58. 10.1186/s13023-019-1014-8 PMC6390548 30808384

[9] Ito N, Hidaka N, Kato H. The pathophysiology of hypophosphatemia. Best Pract Res Clin Endocrinol Metab. 2024;38(2):101851. 10.1016/j.beem.2023.101851 38087658

[10] Ito N, Kang HG, Nishida Y, Evins A, Skrinar A, Cheong HI. Burden of disease of X-linked hypophosphatemia in Japanese and Korean patients: a cross-sectional survey. Endocr J. 2022;69(4):373–383. 10.1507/endocrj.EJ21-0386 34732603

[11] Ito N, Kang HG, Michigami T, et al. Prevalence of comorbid hyperparathyroidism and its association with renal dysfunction in Asian patients with X-linked hypophosphatemic rickets/osteomalacia. Calcif Tissue Int. 2025;116(1):50. 10.1007/s00223-025-01359-9 PMC11903609 40074938

[12] Portale AA, Ward L, Dahir K, et al. Nephrocalcinosis and kidney function in children and adults with X-linked hypophosphatemia: baseline results from a large longitudinal study. J Bone Miner Res. 2024;39(10):1493–1502. 10.1093/jbmr/zjae127 PMC11425691 39151033

[13] DeLacey S, Liu Z, Broyles A, et al. Hyperparathyroidism and parathyroidectomy in X-linked hypophosphatemia patients. Bone. 2019;127:386–392. 10.1016/j.bone.2019.06.025 PMC6836672 31276850

[14] Mӓkitie O, Kooh SW, Sochett E. Prolonged high-dose phosphate treatment: a risk factor for tertiary hyperparathyroidism in X-linked hypophosphatemic rickets. Clin Endocrinol. 2003;58(2):163–168. 10.1046/j.1365-2265.2003.01685.x 12580931

[15] Lecoq AL, Chaumet-Riffaud P, Blanchard A, et al. Hyperparathyroidism in patients with X-linked hypophosphatemia. J Bone Min Res. 2020;3(7):1263–1273. 10.1002/jbmr.3992 32101626

[16] National Institute for Health and Care Excellence . Burosumab. https://www.bnf.nice.org.uk. [2025]. Updated 2025. Accessed 24/09/2025. https://bnf.nice.org.uk/drugs/burosumab/

[17] Lamb YN . Burosumab: first global approval. Drugs. 2018;78(6):707–714. 10.1007/s40265-018-0905-7 29679282

[18] Kyowa Kirin . Kyowa Kirin Announces Approval of Crysvita (Burosumab) for the Treatment of FGF23-Related Hypophosphatemic Rickets and Osteomalacia in Japan. https://www.kyowakirin.com. Accessed 24, 2025. https://www.kyowakirin.com/media_center/news_releases/2019/e20190920_01.html

[19] Michigami T, Kang HG, Namba N, et al. Burosumab treatment of X-linked hypophosphatemia patients: interim analysis of the SUNFLOWER longitudinal, observational cohort study. JBMR Plus. 2024;8(8):ziae079. 10.1093/jbmrpl/ziae079 PMC11250265 39015507

[20] Namba N, Kubota T, Muroya K, et al. Safety and efficacy of burosumab in pediatric patients with X-linked hypophosphatemia: a phase 3/4 open-label trial. J Endocr Soc. 2022;6(5):bvac021. 10.1210/jendso/bvac021 PMC8962727 35356008

[21] Carpenter TO, Whyte MP, Imel EA, et al. Burosumab therapy in children with X-linked hypophosphatemia. New Engl J Med. 2018;378(21):1987–1998. 10.1056/NEJMoa1714641 29791829

[22] Ward LM, Hӧgler W, Glorieux FH, et al. Burosumab vs conventional therapy in children with X-linked hypophosphatemia: results of the open-label, phase 3 extension period. JBMR Plus. 2024;8(1):ziad001. 10.1093/jbmrpl/ziad001 PMC11059996 38690124

[23] Wang K, Zhang R, Chen Z, Bai Y, He Q. Meta-analysis and systematic review: burosumab as a promising treatment for children with X-linked hypophosphatemia. Front Endocrinol. 2024;15:1414509. 10.3389/fendo.2024.1414509 PMC11357961 39211452

[24] Haffner D, Emma F, Seefried L, et al. Clinical practice recommendations for the diagnosis and management of X-linked hypophosphataemia. Nature Rev Nephrol. 2025;21:330–354. 10.1038/s41581-024-00926-x 39814982

[25] Munns CF, Yoo HW, Jalaludin MY, et al. Asia-Pacific consensus recommendations on X-linked hypophosphatemia: diagnosis, multidisciplinary management, and transition from pediatric to adult care. JBMR Plus. 2023;7(6):e10744. 10.1002/jbm4.10744 PMC10241092 37283655

[26] Sandy JL, Simm PJ, Biggin A, et al. Clinical practice guidelines for paediatric X-linked hypophosphataemia in the era of burosumab. J Paediatr Child Health. 2022;58(5):762–768. 10.1111/jpc.15976 35426466

[27] Ali DS, Carpenter TO, Imel EA, et al. X-linked hypophosphatemia management in children: an international working group clinical practice guideline. J Clin Endocrinol Metab. 2025;110(7):2055–2070. 10.1210/clinem/dgaf093 PMC12187519 39960858

[28] Padidela R, Cheung MS, Saraff V, Dharmaraj P. Clinical guidelines for burosumab in the treatment of XLH in children and adolescents: British paediatric and adolescent bone group recommendations. Endocr Connect. 2020;9(10):1051–1056. 10.1530/EC-20-0291 PMC7707830 33112809

[29] Canadian Journal of Health Technologies . Reimbursement Recommendation. Burosumab (Crysvita). https://www.cda-amc.ca. [10/2024]. Updated 10/2024. Accessed September 24, 2025. https://www.cda-amc.ca/sites/default/files/DRR/2024/SR0818_Reassessment_Final_Recommendation.pdf

[30] Thacher TD, Pettifor JM, Tebben PJ, et al. Rickets severity predicts clinical outcomes in children with X-linked hypophosphatemia: utility of the radiographic Rickets Severity Score. Bone. 2019;122:76–81. 10.1016/j.bone.2019.02.010 30772600

[31] Uday S, Shaw NJ, Mughal MZ, et al. Monitoring response to conventional treatment in children with XLH: value of ALP and Rickets Severity Score (RSS) in a real world setting. Bone. 2021;151:116025. 10.1016/j.bone.2021.116025 34052463

[32] Imel EA, Glorieux FH, Whyte MP, et al. Burosumab versus conventional therapy in children with X-linked hypophosphataemia: a randomised active-controlled, open-label, phase 3 trial. Lancet. 2019;393(10189):2416–2427. 10.1016/S0140-6736(19)30654-3 PMC7179969 31104833

[33] Imel EA, Glorieux FH, Whyte MP, et al. Burosumab vs phosphate/active vitamin D in pediatric X-linked hypophosphatemia: a subgroup analysis by dose level. J Clin Endocrinol Metab. 2023;108(11):2990–2998. 10.1210/clinem/dgad230 PMC10583998 37084401

[34] Kubota T, Fukumoto S, Cheong HI, et al. Long-term outcomes for Asian patients with X-linked hypophosphataemia: rationale and design of the SUNFLOWER longitudinal, observational cohort study. BMJ Open. 2020;10(6):e036367. 10.1136/bmjopen-2019-036367 PMC7328740 32601114

[35] Kyowa Kirin Co., Ltd . Prescribing Information: Burosumab. Updated January 2026. Accessed March 14, 2026. https://pins.japic.or.jp/pdf/newPINS/00071730.pdf 10.1186/s13023-026-04267-9

[36] Whyte MP, Fujita KP, Moseley S, Thompson DD, McAlister WH. Validation of a novel scoring system for changes in skeletal manifestations of hypophosphatasia in newborns, infants, and children: the radiographic global impression of change scale. J Bone Miner Res. 2018;33(5):868–874. 10.1002/jbmr.3377 29297597

[37] Colantonio DA, Kyriakopoulou L, Chan MK, et al. Closing the gaps in pediatric laboratory reference intervals: a CALIPER database of 40 biochemical markers in a healthy and multiethnic population of children. Clin Chem. 2012;58(5):854–868. 10.1373/clinchem.2011.177741 22371482

[38] Isojima T, Kato N, Ito Y, Kanzaki S, Murata M. Growth standard charts for Japanese children with mean and standard deviation (SD) values based on the year 2000 national survey. Clin Pediatr Endocrinol. 2016;25(2):71–76. 10.1297/cpe.25.71 PMC4860518 27212799

[39] Wilson SM, Bohn MK, Madsen A, Hundhausen T, Adeli K. LMS-based continuous reference percentiles for 14 laboratory parameters in the CALIPER cohort of healthy children and adolescents. Clin Chem Lab Med. 2023;61(6):1105–1115. 10.1515/cclm-2022-1077 36639844

[40] Pott V, Tietze H, Kanzelmeyer N, et al. LMS-based pediatric reference values for parameters of phosphate homeostasis in the HARP cohort. J Clin Endocr Metab. 2024;109(3):668–679. 10.1210/clinem/dgad597 37850343

[41] Levy-Shraga Y, Levi S, Regev R, et al. Linear growth of children with X-linked hypophosphatemia treated with burosumab: a real-life observational study. Eur J Pediatr. 2023;182(11):5191–5202. 10.1007/s00431-023-05190-y 37707589

[42] Namba N, Ito N, Michigami T, et al. Impact of X-linked hypophosphatemic rickets/osteomalacia on health and quality of life: baseline data from the SUNFLOWER longitudinal, observational cohort study. JBMR Plus. 2024;8(11):ziae118. 10.1093/jbmrpl/ziae118 PMC11470975 39399158

[43] Fukumoto S, Haffner D, Imel EA, et al. Real-world impact of treatment on growth in children with X-linked hypophosphatemia. J Clin Endocrinol Metab. 2026;**111**(9):2513-2524. 10.1210/clinem/dgag136 PMC13466958 41911946

[44] Lim R, Shailam R, Hulett R, et al. Validation of the radiographic global impression of change (RGI-C) score to assess healing of rickets in pediatric X-linked hypophosphatemia (XLH). Bone. 2021;148:115964. 10.1016/j.bone.2021.115964 33878504

[45] Kyowa Kirin . Radiographic Global Impression of Change (RGI-C). [06/2020]. Updated June 2020. Accessed September 24, 2025. https://content-eu-1.content-cms.com/df8c883f-9c2a-4f6f-a99a-9c610535fd9e/dxdam/00/00b5ed2a-b19e-4b1a-9b67-e75725873042/crysvita-rgic.pdf

