# Supplementary material for: Effectiveness of burosumab in children with X-linked hypophosphatemia with low rickets severity: SUNFLOWER analysis
Source: JBMR Plus. 2026 Oct 15;10(12):ziag168. doi: 10.1093/jbmrpl/ziag168 (PMC13732572; doi:10.1093/jbmrpl/ziag168)
Supplement: Supplementary file 1 [file supplementary_materials_ziag168.docx]

**Supplementary Materials**

**Effectiveness of burosumab in children with X-linked hypophosphatemia with low rickets severity: SUNFLOWER analysis**

Hee Gyung Kang, MD, PhD, Toshimi Michigami, MD, PhD, Noriyuki Namba, MD, PhD, Nobuaki Ito, MD, PhD, Takuo Kubota, MD, PhD, Osamu Miyazaki, MD, PhD, Masanori Kanematsu, Chikako Hayashi, PhD, Haruka Ishii, MSc, Seiji Fukumoto, MD, PhD, and Keiichi Ozono, MD, PhD

**Figure S1.** Flowchart of the all-patients analysis and the full 3-yr analysis in the burosumab and non-burosumab groups.

**Figure S2.** Patient flowchart.

Overall, 226 patients participated in the SUNFLOWER study (April 1, 2018, to December 31, 2023). Patients aged >13 yr at enrollment (n = 147) and who received burosumab prior to enrollment (n = 31) (with some overlap, n = 157) were excluded from this interim analysis, leaving 69 patients. Among them, 65 and 4 patients were in the non-burosumab and burosumab groups, respectively, at enrollment. Overall, 44 patients in the non-burosumab group switched to the burosumab group between enrollment and the cutoff date. Therefore, 48 patients were analyzed in the burosumab group and 65 were analyzed in the non-burosumab group.

*Including patients who had not yet reached the 3-yr scheduled visit or who discontinued follow-up during the study (n = 3).

**Patients reassigned from the non-burosumab to the burosumab treatment group in the follow-up period. These patients were subsequently followed up as members of the burosumab group rather than the non-burosumab group.

***Including patients who had not yet reached the 3-yr scheduled visit, who discontinued follow-up during the study, or who discontinued burosumab treatment (n = 15).

**Figure S3.** Results of the all-patients analysis and the full 3-yr analysis in the non-burosumab group. A) Height Z-score, B) ALP Z-score, C) RSS, D) phosphate Z-score, E) 1,25(OH)_2_D, and F) iPTH are shown at enrollment, 3 mo, 6 mo, 1 yr, 2 yr, and 3 yr for the all-patients analysis (irrespective of treatment duration and missing data) and the full 3-yr analysis (without missing data) in the non-burosumab group. The boxes represent the IQR, and the median is indicated by the horizontal line inside each box. The whiskers extend to the most extreme values within 1.5 × IQR. The mean value is indicated by a cross (×), and the circles represent outliers defined as values outside the whiskers (1.5 × IQR). n^†^ indicates the number of patients with available data. Abbreviations: ALP, alkaline phosphatase; BL, baseline; iPTH, intact PTH; IQR, interquartile range; mo, months; RSS, Rickets Severity Score; yr, year.

**Figure S4.** Results of the subgroup analysis by baseline RSS status (RSS <2 or RSS ≥2) in the burosumab group (full 3-yr analysis). A) Height Z-score, B) ALP Z-score, C) RSS, D) phosphate Z-score, E) 1,25(OH)_2_D, and F) iPTH are shown at enrollment, 3 mo, 6 mo, 1 yr, 2 yr, and 3 yr. The boxes represent the IQR, and the median is indicated by the horizontal line inside each box. The whiskers extend to the most extreme values within 1.5 × IQR. The mean value is indicated by a cross (×), and the circles represent outliers defined as values outside the whiskers (1.5 × IQR). n^†^ indicates the number of patients with available data. Abbreviations: ALP, alkaline phosphatase; BL, baseline; iPTH, intact PTH; IQR, interquartile range; mo, month; RSS, Rickets Severity Score; SoB, start of burosumab; yr, year.

**Figure S5.** Results of the subgroup analysis by ALP status (ALP <ULN or ALP ≥ULN) in the burosumab group (full 3-yr analysis). A) Height Z-score, B) ALP Z-score, C) RSS, D) phosphate Z-score, E) 1,25(OH)_2_D, and F) iPTH are shown at the SoB, 3 mo, 6 mo, 1 yr, 2 yr, and 3 yr. The boxes represent the IQR, and the median is indicated by the horizontal line inside each box. The whiskers extend to the most extreme values within 1.5 × IQR. The mean value is indicated by a cross (×), and the circles represent outliers defined as values outside the whiskers (1.5 × IQR). n^†^ indicates the number of patients with available data. Abbreviations: ALP, alkaline phosphatase; BL, baseline; iPTH, intact PTH; IQR, interquartile range; mo, month; RSS, Rickets Severity Score; SoB, start of burosumab; yr, year.

**Supplementary Table**

**Table S1.** Numerical changes from baseline in the primary outcome measures in the non-burosumab group (all-patients analysis and full 3-yr analysis [without missing data]).

|  |  | **All patients** | | | | **Patients treated for the full 3 yr without missing data** | | | |
| --- | --- | --- | --- | --- | --- | --- | --- | --- | --- |
|  |  | **Enrollment**  **(n = 65)** | **1 yr**  **(n = 58)** | **2 yr**  **(n = 34)** | **3 yr**  **(n = 27)** | **Enrollment**  **(n = 10)** | **1 yr**  **(n = 10)** | **2 yr**  **(n = 10)** | **3 yr**  **(n = 10)** |
| Height (Z-score) | n^†^ | 60 | 35 | 20 | 20 | 10 | 10 | 10 | 10 |
|  | Mean ± SD | −1.85 ± 1.21 | −1.87 ± 1.08 | −1.45 ± 1.00 | −1.25 ± 0.96 | −1.36 ± 1.04 | −1.51 ± 0.97 | −1.50 ± 0.97 | −1.39 ± 0.86 |

Here, n indicates the number of patients, and n^†^ indicates the number of patients with available data.

Abbreviations: SD, standard deviation; yr, year.
